# The application of chimeric deep circumflex iliac artery perforator flap for oromandibular reconstruction: A case report

**DOI:** 10.1097/MD.0000000000025458

**Published:** 2021-04-09

**Authors:** Chengyao Zhang, Yi Zeng, Lian Zhou, Xi Tang

**Affiliations:** a Department of Head and Neck Cancer Center; b Department of Clinical Laboratory, Chongqing University Cancer Hospital, Chongqing University, Chongqing, China.

**Keywords:** case report, chimeric flap, deep circumflex iliac artery, mandible reconstruction, squamous cell carcinoma

## Abstract

**Rationale:**

The free fibular flap is considered the gold standard, particularly for a mandibular defect combined with a significant soft tissue defect. However, the fibular flap has the disadvantages of a lack of height for postoperative dental restoration and donor site skin graft if the skin paddle is wider than 5 cm. The larger bone and soft tissue defects tend to be reconstructed using either a scapula or a combination of iliac artery and radial free flap. Few cases involving reconstruction using chimeric deep circumflex iliac artery perforator flap (DCIAPF) for mandibular defect combined with more significant soft tissue defects have been reported due to perforator variations. We successfully performed oromandibular reconstruction using chimeric DCIAPF.

**Patient concerns:**

A 56-year-old male patient was admitted due to “constant pain in the gradually enlarged right lower gingival mass since the previous four months.” The patient had no other obvious symptoms, and no history of diabetes or hypertension was reported. The patient reported long-term smoking and drinking habits.

**Diagnoses:**

Computed tomography (CT) revealed a neoplasm in the right buccal space, which is primarily considered a malignancy. The pathological results of a gingival mass biopsy presented squamous cell carcinoma.

**Interventions:**

No operative contraindications were confirmed after regular tests and examinations were undertaken. The patient underwent a primary extent resection of a 6-cm-long mandible, including mass and suprascapulohyoid neck dissection. The oromandibular defects were then reconstructed with chimeric DCIAPF, simultaneously using the iliac crest bone flap to repair the mandibular lateral segment defect and the skin paddle to repair the intraoral soft tissue defect of 5 × 10 cm.

**Outcomes:**

The total operating time was five and half hours and blood loss was approximately 500 ml. The operation was successful, with no infections or flap loss. Six months postoperatively, CT showed that the iliac crest bone had connected to the alveolar bone of the mandible. The height of the iliac crest bone was sufficient for postoperative dental restoration. The patient healed without obvious complications and no tumor recurrence.

**Lessons:**

Chimeric DCIAPF is an excellent option for mandibular angle or body segment defects combined with significant soft tissue defects.

## 1 Introduction

Mandibular reconstruction followed by tumor ablation remains a considerable challenge for head and neck surgeons. The mandible is an important part of the face in terms of appearance, speech, and chewing. Mandibular resection is necessary for various combinations of bone and/or soft tissue defects. There are 4 common bone sources for microvascular transfer, the radius, scapula, fibula, and iliac crest,^[1–2]^ of which the fibular free flap is currently considered the gold standard and the workhorse of mandibular reconstruction.^[3]^ However, the disadvantages of the fibular flap include a lack of height for dental restoration and placement of the plate.^[4]^ Previously, forearm flaps, groin flaps, or anterolateral thigh flaps^[5]^ were used to reconstruct soft tissues, while the deep circumflex iliac artery (DCIA) bone flap was used for mandibular reconstruction. The chimeric DCIA perforator flap (DCIAPF) provides sufficient height for dental restoration and plate fixation, as well as oromandibular soft tissue reconstruction. However, few cases involving reconstruction with chimeric DCIAPF have been reported. We present a case of a 56-year-old male patient with a reconstructed DCIAPF with iliac crest for an oromandibular composite defect following tumor ablation, and provide a brief literature review on the subject.

## 2 Case report

A 56-year-old male patient was admitted to our hospital with complaints of constant pain in the gradually enlarged right lower gingival mass for 4 months prior to consultation. The patient reported long-term smoking and drinking habits.

**Figure 1:**
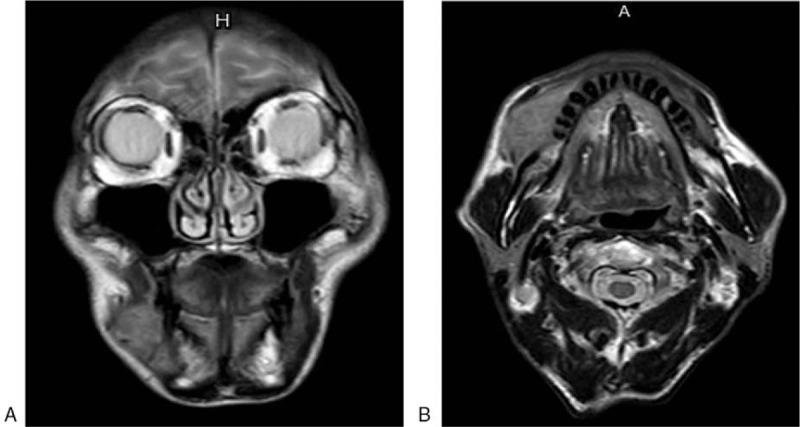
Magnetic resonance images of the neck presented a mass shadow on the right cheek with no clear boundary and a size of approximately 4.0 × 2.2 cm. (A. *axial view*; B. *coronal view*).

A 2.0 × 3.0-cm cauliflower-shaped mass was located at the gingiva of the fourth tooth of the right mandible, which bled easily upon palpation. The surrounding mucosa was observed to be without oral lesions. The cervical lymph nodes were not enlarged upon palpation. A biopsy of the gingival mass was carried out, and the pathological result presented squamous cell carcinoma. Magnetic resonance images (Fig. 1) of the neck presented a mass shadow on the right cheek with no clear boundary and a size of approximately 4.0 × 2.2 cm. T1W showed an equal signal, and T2W and T2W fat pressure showed a high signal. The boundary with the right inferior gingiva was not clear, and was adjacent to the right maxilla and the right side of the mandible abnormal signal. Positron emission tomography-computed tomography (PET-CT) images of the neck showed a soft tissue density shadow in the right lower gingiva measuring approximately 1.8 × 3.2 cm. Metabolism was increased. Multiple enlarged lymph nodes were identified at level II of the right side of the neck, and metabolism was slightly increased.

**Figure 2:**
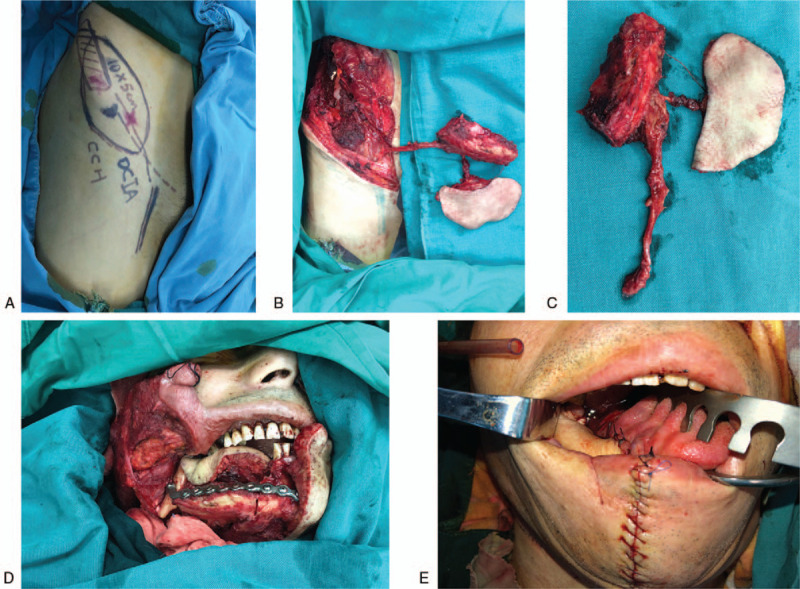
Intraoperative pictures, A: Surgical design and preoperative mark of perforators; B DCIAPF harvested; C: DCIAPF after pedicle cut off; D: Place the flap to the oromandibular defect, and fix the iliac crest bone with mandible; E: Intraoral soft tissue defect reconstructed with skin paddle of DCIAPF.

After a preoperative discussion with the patient to establish a surgery plan, the patient consented to the use of chimeric DCIAPF to reconstruct the oromandibular defect after tumor ablation. The perforators of DCIA were marked using Duplex-Doppler ultrasound preoperatively (Fig. 2A). The patient was positioned supine, and general anesthesia was administered through nasal intubation. Extensive resection of the tumor was performed after emptying the cervical lymph nodes at levels I–III ipsilateral to the tumor, and a 6-cm-long mandibular body segment and intraoral mucosal soft tissue composite defects were created.

## 3 Techniques for harvesting the chimeric deep circumflex iliac artery perforator flap

According to the preoperative design, a median incision of the skin paddle was made to reach the muscle layer of the abdominal wall. The skin paddle was carefully separated from the median to the lateral side. The skin paddle design was adjusted after the reliable perforator was exposed. The whole skin paddle was evaluated only with the perforator connected, and the dominated perforator was dissected retrogradely to the iliac crest in the muscular layer of the abdominal wall. The branches of nutritional abdominal muscles were ligated. The iliac crest was incised to the midpoint of the inguinal ligament to expose the crest, external oblique muscle, and inguinal ligament. The muscle attached to the inguinal ligament was removed to expose the femoral vessels. The origin of the deep circumflex iliac vascular bundle was identified at the junction of the external iliac artery and femoral artery. The ilioinguinal nerve crossing was protected, and the abdominal wall was incised layer by layer along the vascular bundle. The deep circumflex iliac vessels sent out several small perforating branches to nourish the internal iliac bone plate after passing through the anterior superior iliac spine (ASIS). The lateral femoral cutaneous nerve was isolated and protected. The abdominal muscle was cut along the internal plate of the iliac bone. The muscle sleeve, measuring approximately 1 cm, surrounding the blood vessel was reserved and connected to the iliac bone. The vascular pedicle was freed until it was connected to the cutaneous island vessel. The tensor fascia lata and gluteus medius attached to the external plate of the iliac bone were stripped to avoid soft tissue on the surface. According to the mandible defect, iliac osteotomy was performed with a micro dynamic bone saw from 2 cm behind the ASIS. The bone and skin island was completely freed from the donor site (Fig. 2B). The blood supply of the iliac crest bone and skin paddle was assessed subsequently, and the pedicle was removed (Fig. 2C). after the vessel preparation of the recipient area was completed. The abdominal wall was tightly sutured in layers to avoid an abdominal hernia.

The iliac bone was placed with the mandibular defect (Fig. 2D)., and the skin paddle was placed with the intraoral defect (Figure 2E). Subsequently, the iliac crest bone was fixed to the mandible. The deep circumflex iliac vessels were then anastomosed to the ipsilateral neck vessels.

Postoperative pathological examination showed squamous cell carcinoma of the right lower gingiva, well-differentiated, with invasion of the lamina propria. No tumor involvement was found in the mandible, no vascular tumor thrombus was found, and nerve invasion was negative (−). No metastasis was found in the regional lymph nodes (LN) (0/40): LN 0/10 in level I, LN 0/18 in level II, and LN 0/12 in level III. Postoperative diagnosis was right lower gingival squamous cell carcinoma (T2N0M0), according to tumor node metastasis classification. There was no follow-up treatment.

**Figure 4:**
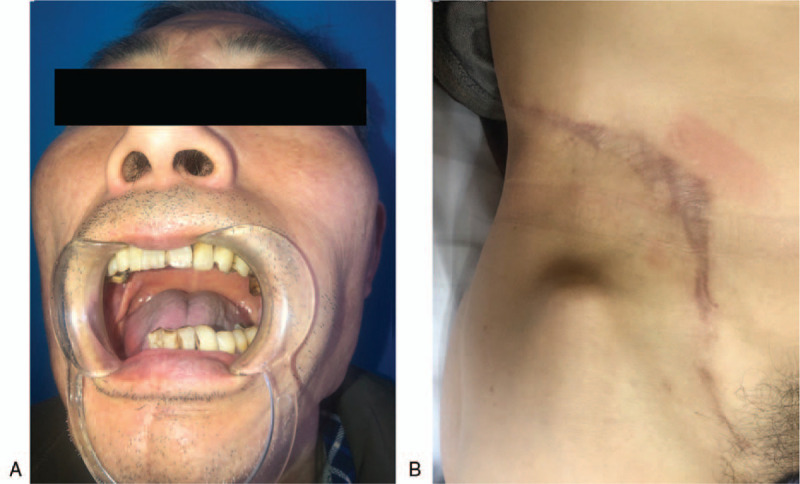
12-month follow-up after surgery: A: The range of mouth opening is about 2.5 cm; B: The appearance of donor-site. ^*∗*^*DCIAPF =* deep circumflex iliac artery perforator flap.

The patient is currently under a postsurgical 16-month regular follow-up and is in good health without recurrence or any evidence of clinical metastasis. The donor site incision healed well without abdoninal hernia (Fig. 4B). The flap in the oral cavity healed well. The range of postoperative mouth opening is about 2.5 cm (Fig. 4A), no limitation of tongue movement. University of Washington Quality of Life Questionnaire (UW-QOL) score is 1090. No limitation of hip movement at donor site. The Harris Hip Score after operation is more than 95. The patient returned to normal daily life without complaints of any long-term complications. The patient was satisfied with the effectiveness of the surgery.

## 4 Discussion

There are many etiologies of mandibular defects, including tumors, trauma, osteoradionecrosis, and congenital deformities,^[6]^ of which tumors are the most common. Mandibular reconstruction followed by tumor ablation remains a considerable clinical challenge. The mandible is an important area of the face in terms of appearance, speech, chewing, and swallowing. Mandibular resection results in various combinations of bone and soft tissue defects. There are 4 common sources of bone for microvascular transfer: the radius, scapula, fibula, and iliac crest; of these, the fibular free flap is currently considered the gold standard as well as the workhorse of mandibular reconstruction.^[7]^ However, the fibular flap has the following disadvantages:

1. a lack of height for dental restoration and placement of the plate. However, with the use of a double-barrel free fibular flap,^[8]^ the required height for reconstruction of the mandibular defect can be obtained.
2. A longer fibula than needed for reconstruction must be excised to obtain an adequately long vascular pedicle.
3. A skin graft is needed while carrying a larger skin paddle. The iliac crest free flap provides a large volume of well-vascularized bone, has structural similarity to the mandibular body, and is more straightforward to reshape than the fibula flap.^[9]^

However, the widespread use of DCIA flaps is limited by the unnecessary bulk of the “obligatory muscle cuff” and the tethering of the skin to the bone, which renders soft tissue placement in complex oromandibular reconstructions rather challenging. As clinical anatomic research into DICA perforators has increased, a technique without the “obligatory muscle cuff”^[10]^ makes the widespread use of DCIAPF possible. Bergeron et al^[11]^ reported that an average of 1.6 DCIA perforators were present in 92% of specimens; in other words, the skin paddle nourished by the DCIA perforators can be harvested regularly if needed. Furthermore, Zheng et al^[12]^ described 3 types of DCIA perforators, including the abdominal muscular branches, the iliac osteomuscular branches, and the terminal musculocutaneous perforator. The blood supply of the skin and bone of conventional DCIA osteomusculocutaneous flaps is from the several minute osteomusculocutaneous perforators of DCIA.

Chimeric DCIAPF can provide sufficient bone height for dental restoration and plate fixation, as well as sufficient soft tissue for oromandibular soft tissue reconstruction. The average size of the skin paddle reported by Zheng^[5]^ is 9.6 × 12.8 cm, which is much larger than the conventional skin paddle size of the fibular flap. Moreover, the donor site can be closed directly. The chimeric DCIAPF does not have to carry a large amount of abdominal muscle, which makes the skin paddle less bulky and reduces the incidence of abdominal hernia. It also has a great degree of mobility between the bone component and skin paddle when used for composite oromandibular defect reconstruction. It is flexible to allow the adjustment of the skin paddle to the required location.

**Figure 3:**
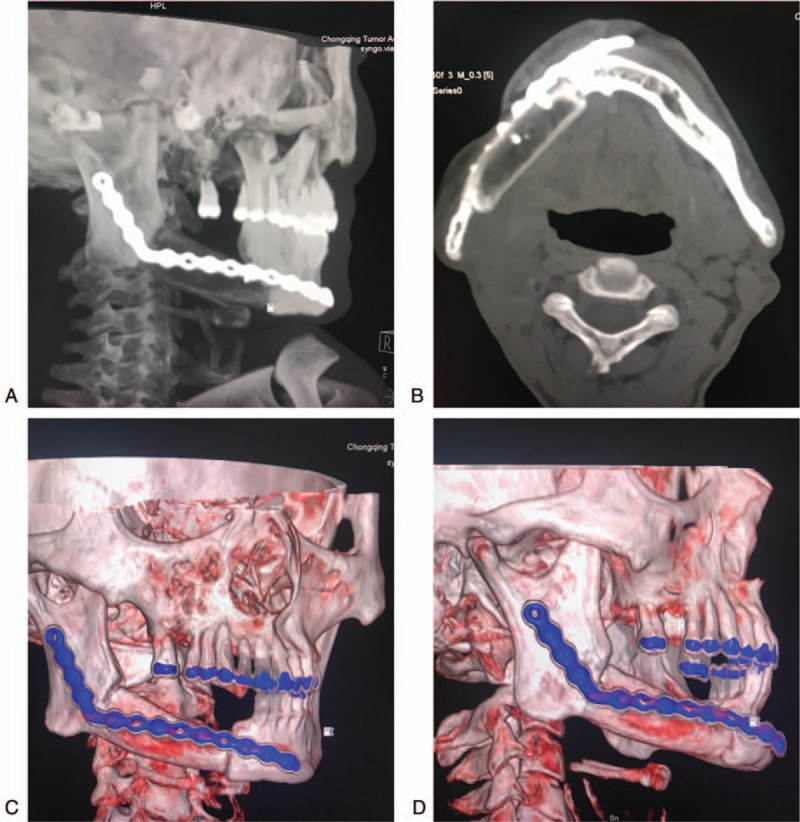
6-month follow-up after surgery: Three-dimensional reconstruction computed tomography of mandible showed the iliac crest bone was connected with the alveolar bone of the mandible, the height of the iliac crest bone was sufficient.

In our case, the composite oromandibular defect included a 6-cm-long mandibular body defect and a 5 × 10-cm intraoral soft tissue defect, which was the perfect indication for chimeric DCIAPF. The shape of the iliac crest bone matched the mandibular defect well, and the height of the iliac crest bone also met the need for postoperative dental restoration (Fig. 3), which largely improves quality of life for the patient postoperatively. The size of the skin paddle is large enough to reconstruct intraoral soft tissue defects, while the donor site can be closed directly without any complications.

Six months postoperatively, CT of the patient in this case study showed that the iliac crest bone was connected to the alveolar bone of the mandible. The iliac crest bone matched the original mandible well, and the height of the iliac crest bone was sufficient for postoperative dental restoration. These follow-up results of chimeric DCIAPF indicated that this case had been a success. The patient healed without obvious complications and with no tumor recurrence. Extensive resection is a crucial step in avoiding tumor recurrence postoperatively. Many tumor recurrences result from conservatively removing the tumor, resulting in the margin potentially not being tumor-free. Various reconstruction techniques allow the removal of tumors as extensively as needed to decrease the incidence of tumor recurrence.

There are several important techniques for harvesting chimeric DCIAPF.

1. Marking the perforator preoperatively is important, as it saves time when harvesting the flap.
2. The marking of perforators may not be accurate; thus, the skin paddle design should be adjusted according to the real location of the perforators in the case of flap failure.
3. For patients to tie their belt as they used to, 2 cm of the ASIS should be preserved.^[13]^
4. Some muscle cuff should be carried with the perforators to avoid injuring them.

## 5 Conclusion

This case suggests that chimeric DCIAPF is an excellent option for mandibular lateral or anterior segment defect combined with significant soft tissue defects. However, more cases are needed to evaluate the rate of success and clinical effects.

## Author contributions

**Conceptualization:** Lian Zhou, Xi Tang.

**Data curation:** Yi Zeng.

**Formal analysis:** Lian Zhou.

**Investigation:** Yi Zeng.

**Writing – original draft:** Chengyao Zhang.

**Writing – review & editing:** Xi Tang.
